# The spike glycoprotein genes of porcine epidemic diarrhea viruses isolated in China

**DOI:** 10.1186/s13567-021-00954-6

**Published:** 2021-06-15

**Authors:** Pei-Hua Wang, Ya-Qian Li, Yuan-Qing Pan, Yan-Yan Guo, Fan Guo, Rui-Zhu Shi, Li Xing

**Affiliations:** 1 Institutes of Biomedical Sciences, Shanxi University, 92 Wucheng Road, Taiyuan, 030006 Shanxi China; 2 Shanxi Provincial Key Laboratory of Medical Molecular Cell Biology, Shanxi University, 92 Wucheng Road, Taiyuan, 030006 China; 3 Shanxi Provincial Key Laboratory for Prevention and Treatment of Major Infectious Diseases, 92 Wucheng Road, Taiyuan, 030006 China

**Keywords:** Porcine epidemic diarrhea virus (PEDV), Phylogenetic analysis, Recombination, B cell epitope, Spike glycoprotein, China

## Abstract

The porcine epidemic diarrhea virus (PEDV) causes a highly contagious disease in pigs, which is one of the most devastating viral diseases of swine in the world. In China, PEDV was first confirmed in 1984 and PEDV infections occurred sporadically from 1984 to early 2010. From late 2010 until present, PEDV infections have swept every province or region in China. In this study, we analyzed a total of 186 full-length spike genes and deduced proteins of all available complete genomes of PEDVs isolated in China during 2007–2019. A total of 28 potential recombination events were identified in the spike genes of PEDVs in China. Spike gene recombination not only expanded the genetic diversity of PEDVs in the GII genogroup, but also resulted in the emergence of a new evolutional branch GI-c during 2016–2018. In addition, comparative analysis of spike proteins between GI-a prototype virulent CV777 and GII strain AJ1102 reveals that the amino acid variations could affect 20 potential linear B cell epitopes, demonstrating a dramatic antigen drift in the spike protein. These results provide a thorough view of the information about the genetic and antigenic diversity of PEDVs circulating in China and therefore could benefit the development of suitable strategies for disease control.

**Supplementary Information:**

The online version contains supplementary material available at 10.1186/s13567-021-00954-6.

## Introduction

Porcine epidemic diarrhea (PED) is caused by porcine epidemic diarrhea virus (PEDV) [1]. PED is characterized by severe watery diarrhea, vomiting, anorexia, dehydration, and weight loss in pigs [1]. PEDV is highly contagious and infects pigs of all ages, but the condition is especially severe in piglets, with morbidity and mortality often reaching 100% [2, 3]. PED was first reported in England in 1971, but PEDV was isolated for the first time in Belgium in 1978 and designated as prototype coronavirus virulent CV777 [4]. Thus far, PED has been reported in swine-farming countries in Europe, Asia, North America, and South America as one of the most devastating viral diseases of swine in the world, leading to enormous economic loss in the global pork industry [1, 5–8].

PEDV is a large enveloped RNA virus. It is a member of the genus *Alphacoronavirus* within the *Coronaviridae* family in the order *Nidovirales* [4]. The PEDV genome RNA is approximately 28 kb in length with a 5′-cap and a 3′-polyadenylated tail. It contains at least 7 open reading frames (ORF1a, ORF1b, and ORF2-6) [9]. ORFs 1a and 1b encode for viral non-structural proteins (nsps). ORF1a translation produces a replicase polyprotein 1a (ppla) while a -1 ribosomal frame shift (RFS) at the 3′-end of ORF1a allows the translation of ORF1b through the production of the replicase polyprotein 1ab (pp1ab). The ppla and pplab are processed during translation by internal proteases into 16 nsps that are mainly responsible for the regulation of transcription, translation, and viral RNA synthesis in the host cells [10]. The remaining ORFs are located in the 3′-proximal region of viral genome and encode four structural proteins including the 150–220 kDa glycosylated spike (S) protein, 20–30 kDa membrane (M) protein, 7 kDa envelope (E) protein, 58 kDa nucleocapsid (N) protein, and one accessory protein [9, 11].

The N protein interacts with viral genomic RNA and then forms the helical nucleocapsid during viral particle assembly [12]. The M protein is also required for the assembly process and can elicit the production of antibodies [13, 14]. The E protein plays an important role in the budding of the viral particle [15]. The accessory protein encoded by ORF3 is thought to serve as an ion channel and can affect virus production and virulence [16, 17].

The S protein of PEDV is the major envelope type I glycoprotein of the virion [18]. It is located on the surface of viral particles and interacts with the cellular receptor during virus entry into host cells [19]. The S protein forms a homotrimer complex on the surface of coronavirus virion and is responsible for the virus binding to specific cellular receptor [20]. During virus entry into host cell, the S protein monomer is usually cleaved into two subunits, S1 and S2 subunits. The S1 subunit binds the cellular receptor, which is followed by S2 subunit-mediated fusion of viral and cellular membranes [20, 21]. There is an extra domain (named Domain 0, D0) located at the N terminus of S1subunit of PEDV S protein, which preferentially binds to the sialic acid and modulates the host cell tropism of PEDV [22, 23]. The monomer S protein of PEDV prototype CV777 contains 1383 amino acid (aa) residues. The cryo-electron microscopy (cryo-EM) structure of this protein in the prefusion conformation was resolved at a resolution of 3.1 Å using the soluble ectodomain [24]. The dissociated S1 subunit trimers adopts a conformation differing from that observed in the intact spike proteins, suggesting that this subunit undergoes conformational rearrangements during virus entry [24].

The PEDV S protein is considered to be a major virulence protein [25, 26]. The S1 subunit that contains D0 and S1 domain has been shown to be a determinant part of S protein for viral virulence [27]. Deletion of D0 can attenuate viral virulence [28, 29] while the variants containing D0-deletion remain capable to enter into the host cells in vivo and in vitro [30, 31]. PEDV S protein is also a major protein antigen that elicits the production of neutralizing antibodies [22, 32–34]. Analysis of S1 subunit of PEDV pathogenic strain GDU S protein reveals that both the N-terminal sialic acid binding domain (Domain 0) and the C-terminus of S1 subunit contain neutralizing epitopes [22]. Two neutralizing monoclonal antibodies (NmAbs) E10E-1–10 and P4B-1 can recognize epitopes at amino acids (aa) 435–485 and aa 575–639 of PEDV Pintung 52 (PEDV-PT) S protein, respectively [33]. NmAb 2C10 specifically binds to an epitope at aa 1368–1374 of the PEDV CV777 S protein [35]. In addition, multiple neutralizing linear B cell epitopes have been found at aa 744–774 of S proteins from either PEDV CV777 [36] or CO13 [37]. Therefore, the S protein or its S1 subunit are the major targets in vaccine research [38–40]. In addition, the S protein of coronaviruses is associated with viral growth adaptation and attenuation [41]. Thus, S gene is always employed in determining the genetic relatedness among PEDV isolates or in developing diagnostic assays [38, 42–45].

The recombination of coronavirus S genes has been reported. For example, novel variants of canine enteric coronavirus (CCoV), an alphacoronavirus infecting dogs, have been found to result from the S gene recombination during virus infection [46, 47]. Li et al. also reported a PEDV strain CH/HNQX-3/14 (GenBank: KR095279.1) that arose from the natural recombination between an attenuated vaccine strains (CV777 or DR13) and a pandemic variant (CH/ZMDZY/11) [48]. This recombination occurred not only in structural protein-coding region (S and N genes) but also in non-structural protein-coding region (pp1a gene and ORF3) [48]. PEDV TCN/Liaoning25/2018 (GenBank: MK796238.1) was another natural recombinant strain that contains the S gene from the highly pathogenic CH/GDZQ/2014 strain (GenBank:KM242131.1) and the remaining genomic regions from the low pathogenic vaccine isolate SQ2014 (GenBank: KP728470.1) [49].

In China, PED was first reported in Shanghai in 1973 and the causative agent PEDV was confirmed in 1984 [50]. From 1984 to early 2010, PEDV infections in China occurred in pig farms in a sporadic or regional way, thus there were no large-scale outbreaks [51, 52]. In the early 1990s, a vaccine containing the inactivated prototype CV777 strain was successfully developed in China and has been widely used in pig farms [50]. However, in late 2010, PED epidemics suddenly occurred in major pig-producing provinces in both vaccinated and non-vaccinated herds, from which highly pathogenic variants of PEDV were identified in China [53]. Since then, severe PEDV epidemics have swept every province or region in China [3, 54–57]. At present, PED still remains to be a devastating threat to swine industry of China [50].

In this study, we reported the characteristics of full-length S genes and proteins of PEDV variants isolated in China during 2007–2019. We also found 28 recombination events in S genes. The results will provide helpful information for developing the suitable strategies for disease control in China.

## Materials and methods

### Dataset

A total of 186 complete genomic sequences of PEDVs that were isolated in China from 2007 to 2019 were retrieved from GenBank. The full-length S protein genes were aligned with Clustal Omega [58] and then analyzed for phylogenetic tree construction and recombination event. The viruses in this report were identified by their GenBank ID, name, country, and year of collection (in a format as GenBank accession number: virus name/country-year of collection). In addition, the sequences of 4 more PEDV strains including virulent CV777 (GenBank ID: AF353511.1), virulent DR13 (GenBank ID: JQ023161.1), and attenuated DR13 (GenBank ID: JQ023162.1) that were isolated in Belgium and South Korea, respectively, and attenuated CV777 (GenBank ID: KT323979) were also included as references in the phylogenetic tree construction and recombination analysis.

### Phylogenetic analysis

A total of 190 sequences of PEDV full-length S genes (186 isolated in China plus 4 reference strains) were used to generate neighbor-joining phylogenetic trees with the MEGA-X software [59, 60]. The phylogenetic inference was tested with the bootstrap method with 1000 replications. Bootstrap values greater than 70% were indicated.

### Recombination analysis

The recombination events in the full-length S protein genes were determined by an automatic approach using the RDP4 software package [61]. The potential recombination events were identified by each of 7 algorithms (RDP, GENECONV, Bootscan, MaxChi, Chimaera, SiScan, and PhylPro) embedded in the RDP4 package.

### Prediction of linear B cell epitopes

The linear B cell epitopes on the S protein of PEDV were predicted using BepiPred-2.0 server [62]. The BepiPred-2.0 server is running under IEDB (the immune epitope database) and predicts B-cell epitopes from a protein sequence, using a Random Forest algorithm trained on epitopes and non-epitope amino acids determined from crystal structures. The residues with scores above the threshold value that was set at 0.5 were predicted to be part of an epitope and colored in yellow on the graph (Y-axes depicts residue scores and X-axes indicates residue positions in the sequence). Only conserved fragments of at least 5 amino acid residues that were predicted as potential epitopes by BepiPred-2.0 were taken into consideration in this study.

### Three-dimensional structure prediction

Tertiary structure modeling of individual regions of PEDV S protein including domain 0, S1 domain, and S2 domain was carried out using I-TASER (Iterative Threading ASSEmbly Refinement) server [63–65]. I-TASER is a hierarchical approach to protein structure prediction and structure-based function annotation. It first identifies structural templates from the RCSB protein data bank (PDB) by multiple threading approach LOMETS, with full-length atomic models constructed by iterative template-based fragment assembly simulations.

### Statistical analysis

The statistical analysis was performed with Excel 2019. Data points were reported as the mean ± standard deviation (SD). The student’s *t*-test was used to compare two sets of data.

## Results

### Phylogenetic analysis of full-length S genes of PEDVs isolated in China

**Figure 1:**
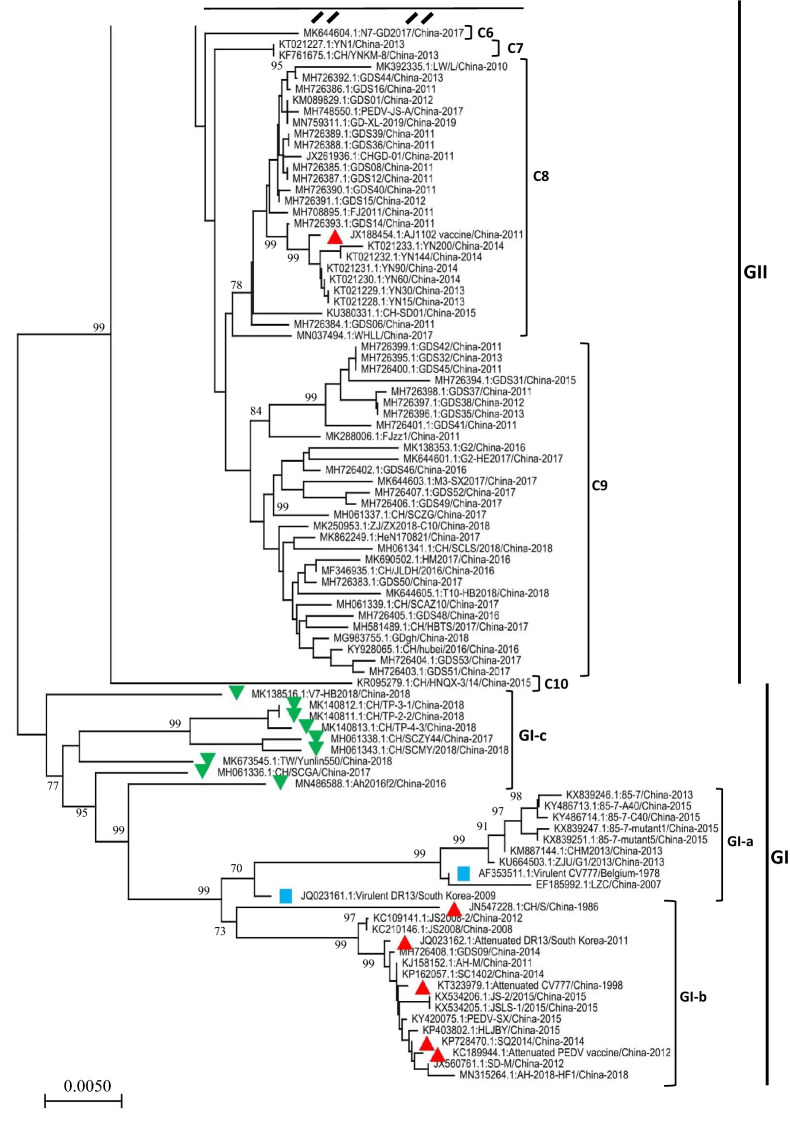
**The phylogenetic tree based on the full‐length spike gene of PEDVs isolated in China during 2007–2019 (continue on Additional file ****1**). Multiple-sequence alignments were performed using Clustal Omega server and the phylogenetic tree was constructed from the aligned nucleotide sequences by using the neighbor-joining method in the MEGA-X software [59, 60]. The numbers at each branch represent bootstrap values greater than 70% of 1000 replicates. The scale bars indicate the number of 0.005 inferred substitutions per site. 4 more PEDV strains including virulent CV777 (AF353511.1), virulent DR13 (JQ023161.1), attenuated CV777 (KT323979), and attenuated DR13 (JQ023162.1) were also included as references. The blue squares indicate the virulent strains isolated outside of China. The red triangles indicate the cell culture-adapted vaccine strains or the pandemic strains used to make vaccines. The green upside-down triangles indicate the GI-c viral isolates.

Phylogenetic analyses using the whole-genome or some individual genes were performed to determine the variations and relationships of PEDV isolates [66, 67]. The partial or full-length S gene are known to be suitable loci in the analysis of genetic relatedness and molecular epidemiology of PEDV [42–44]. To thoroughly understand the variation and evolution of PEDVs in China, we constructed a phylogenetic tree based on the full-length S genes of 190 PEDV strains, 186 of which were isolated in China from 2007 to 2019. The prototype strains (virulent CV777 and virulent DR13) and the cell-culture-adapted vaccine strains (attenuated CV777 and attenuated DR13) are included in the analysis as references. As shown in Figure 1 and Additional file 1, the PEDV strains isolated in China can be classified into two genogroups, i.e*.* GI (classical) and GII (variant). GI genogroup can be further divided into 3 subgroups: GI-a, GI-b, and GI-c. G1a subgroup includes the prototype PEDV strains virulent CV777 and virulent DR13, and 8 Chinese strains including LZC, ZJU/G1 and CHM2013 (Figure 1). GI-b subgroup consists of 3 cell-culture-adapted vaccine strains (attenuated CV777, KT323979.1; attenuated DR13, JQ023162.1; and attenuated PEDV vaccine, KC189944.1) and 13 pandemic classical strains (CH/S, JS2008, JS2008-2, GDS09, AH-M, SC1402, JS-2/2015, JSLS-1/2015, PEDV-SX, HLJBY, SQ2014, SD-M, and AH-2018-HF1). GI-c subgroup emerged as a new evolutionary branch and consists of 9 PEDV strains that were isolated during 2016–2018. These viral isolates are genetically more distant from those in either GI-a or GI-b subgroups. The most of PEDV strains that were isolated in China during 2007–2019 belong to the GII genogroup, which can be further subdivided into 10 clusters (C1-C10) (Figure 1 and Additional file 1).

### Recombination of S genes of PEDVs isolated in China

**Figure 2:**
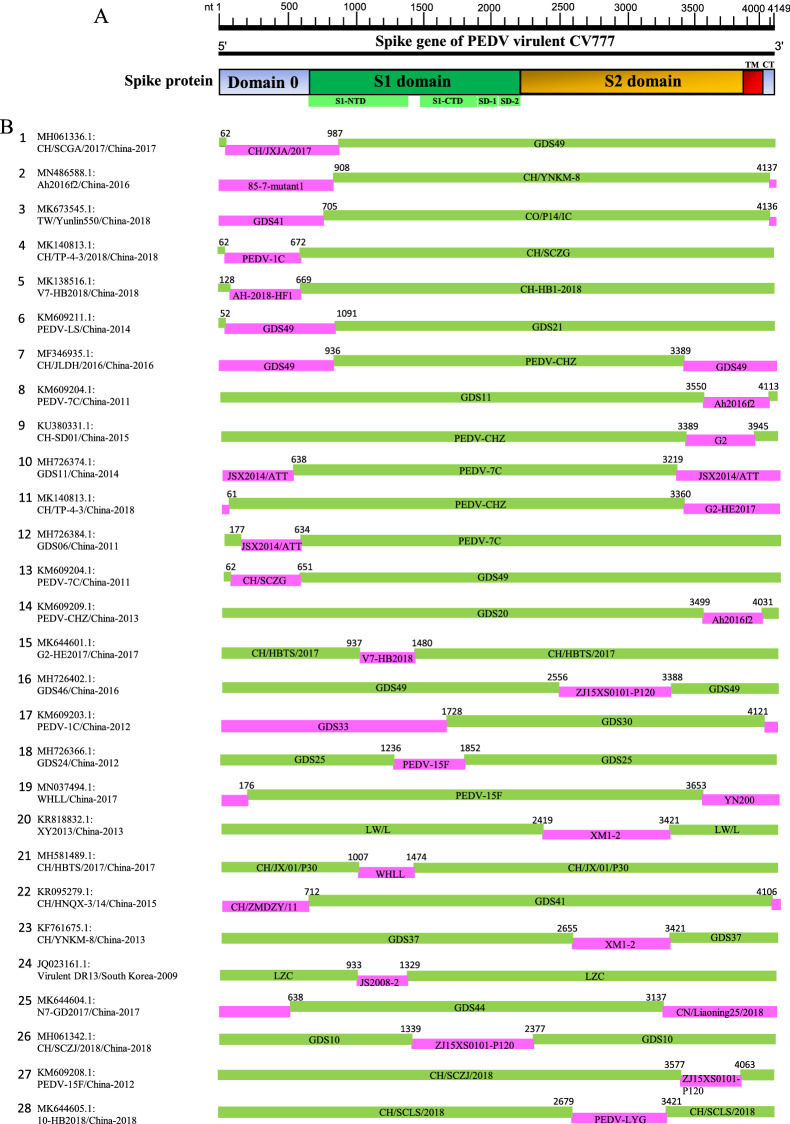
**S gene recombination.** (A) Diagram showing the full-length S gene of PEDV virulent CV777 and the corresponding regions encoding Domain 0, S1 domain, S2 domain, transmembrane domain (TM), and a short cytoplasmic tail (CT). The S1 domain is composed of four subdomains including S1-NTD (S1 N-terminal domain), S1-CTD (S1 C-terminal domain), SD-1, and SD-2. (B) Schematic representation of potential recombination events listed in Table 1. The recombination event serial number and the description of potential recombinants (GenBank ID: virus name/country-collection year) are show on the left. The filled pink and green blocks represent the DNA regions from minor or major parent viruses, respectively. The numbers on the top of filled green blocks indicate the nucleotide positions of breakpoints relative to the spike gene sequence of corresponding recombinant viruses on the left.

In the full-length S gene-based phylogenetic tree, a set of viral isolates that emerged from 2016 to 2018 are sorted into a new subgroup G1-c (Figure 1), implying that the emergence of new PEDV variants will continue and that the virulence-determining S gene may undergo rapid mutation or natural recombination. To identify the recombination events within the S gene of PEDV isolates in China, the full-length S genes included in phylogenetic tree construction (Figure 1) were analyzed using the Recombination Detection Program 4 (RDP4), in which multiple recombination detection methods including RDP, GENECONV, Bootscan, MaxChi, Chimaera, SiScan, and PhylPro were embedded [61]. The recombination analysis reveals that most of PEDV isolates in China seem to arise from 28 potential recombination events in S gene (Table 1, Figure 2). In GI-c subgroup, all of the 9 PEDV isolates (Figure 1) were found to be involved in recombination (Table 1, Figure 2), of which the virus TW/Yunlin550 (GenBank: MK673545.1) has been suggested to arise from the recombination between a Taiwan PEDV GII strain MN and a wild-type GI-a strain virulent DR13 [68]. These results demonstrate that the recombination of S gene is driving the genetic variability of emerging viral isolates in China.

**Table 1:** **Identification of 28 potential recombination events in the spike gene of PEDVs isolated in China during 2007–2019 using RDP4 software package** [61]

Recombination Event serial number	Recombinant		Major parent		Minor parent		Detection methods						
	GenBank ID: Virus name/Country-Year	Geno-group	GenBank ID: Virus name/Country-Year	Geno-group	GenBank ID: Virus name/Country-Year	Geno-group	R	G	B	M	C	S	T
1	MH061336.1:CH/SCGA/2017/China-2017	**GI-c**	MH726406.1:GDS49/China-2017	GII-C5	MF375374.1:CH/JXJA/2017/China-2017	GII-C9	+	+	+	+	+	+	+
2	MN486588.1:Ah2016f2/China-2016	**GI-c**	KF761675.1:CH/YNKM-8/China-2013	GII-C3	KX839247.1:85–7-mutant1/China-2015	**GI-a**	+	+	+	+	+	+	+
3	MK673545.1:TW/Yunlin550/China-2018	**GI-c**	KU558702.1:CO/P14/IC/China-2013	GII-C1	MH726401.1:GDS41/China-2011	GII-C9	+	+	+	+	+	+	+
4	MK140813.1:CH/TP-4–3/2018/China-2018	**GI-c**	MH061337.1:CH/SCZG/China-2017*	GII-C1	KM609203.1:PEDV-1C/China-2012*	GII-C9	+	+	+	+	+	+	+
5	MK138516.1:V7-HB2018/China-2018	**GI-c**	MK606368.1:CH-HB1-2018/China-2018	GII-C5	MN315264.1:AH-2018-HF1/China-2018	**GI-b**	+	+	+	+	+	+	+
6	KM609211.1:PEDV-LS/China-2014	GII-C3	MH726371.1:GDS21/China-2014*	GII-C5	MH726406.1:GDS49/China-2017*	GII-C9	+	+	−	+	+	+	+
7	MF346935.1:CH/JLDH/2016/China-2016	GII-C9	KM609209.1:PEDV-CHZ/China-2013	GII-C1	MH726406.1:GDS49/China-2017	GII-C9	+	+	+	+	+	+	+
8	KM609204.1:PEDV-7C/China-2011	GII-C1	MH726374.1:GDS11/China-2014*	GII-C6	MN486588.1:Ah2016f2/China-2016*	**GI-c**	+	+	−	+	+	+	+
9	KU380331.1:CH-SD01/China-2015	GII-C8	KM609209.1:PEDV-CHZ/China-2013	GII-C1	MK138353.1:G2/China-2016*	GII-C9	+	+	+	+	+	+	+
10	MH726374.1:GDS11/China-2014	GII-C6	KM609204.1:PEDV-7C/China-2011	GII-C1	MH056657.1:JSX2014/ATT/China-2018	GII-C1	+	+	+	+	+	+	+
11	MK140813.1:CH/TP-4–3/China-2018	**GI-c**	KM609209.1:PEDV-CHZ/China-2013	GII-C1	MK644601.1:G2-HE2017/China-2017*	GII-C9	+	+	+	+	+	+	+
12	MH726384.1:GDS06/China-2011	GII-C8	KM609204.1:PEDV-7C/China-2011	GII-C1	MH056657.1:JSX2014/ATT/China-2018	GII-C1	+	+	+	+	+	+	+
13	KM609204.1:PEDV-7C/China-2011	GII-C1	MH726406.1:GDS49/China-2017	GII-C9	MH061337.1:CH/SCZG/China-2017*	GII-C1	+	+	+	+	+	+	+
14	KM609209.1:PEDV-CHZ/China-2013	GII-C1	MH726363.1:GDS20/China-2012*	GII-C1	MN486588.1:Ah2016f2/China-2016*	**GI-c**	−	+	+	+	+	−	+
15	MK644601.1:G2-HE2017/China-2017	GII-C9	MH581489.1:CH/HBTS/2017/China-2017*	GII-C9	MK138516.1:V7-HB2018/China-2018*	**GI-c**	+	+	−	+	+	+	+
16	MH726402.1:GDS46/China-2016	GII-C9	MH726406.1:GDS49/China-2017*	GII-C9	MK409659.1:ZJ15XS0101-P120/China-2016*	GII-C1	−	+	−	+	+	+	+
17	KM609203.1:PEDV-1C/China-2012	GII-C1	MH726369.1:GDS30/China-2014*	GII-C6	MH726379.1:GDS33/China-2014*	GII-C2	+	+	+	+	+	+	+
18	MH726366.1:GDS24/China-2012	GII-C4	MH726365.1:GDS25/China-2013*	GII-C2	KM609208.1:PEDV-15F/China-2012*	GII-C3	−	−	−	+	+	+	+
19	MN037494.1:WHLL/China-2017	GII-C8	KM609208.1:PEDV-15F/China-2012	GII-C3	KT021233.1:YN200/China-2014	GII-C8	−	−	−	+	+	+	+
20	KR818832.1:XY2013/China-2013	GII-C4	MK392335.1:LW/L/China-2010*	GII-C8	KX812523.1:XM1-2/China-2016*	GII-C1	−	+	+	+	+	+	+
21	MH581489.1:CH/HBTS/2017/China-2017	GII-C9	KX058033.1:CH/JX/01/P30/China-2014	GII-C1	MN037494.1:WHLL/China-2017	GII-C8	−	−	−	−	−	+	−
22	KR095279.1:CH/HNQX-3/14/China-2015	GII-C10	MH726401.1:GDS41/China-2011*	GII-C9	KC196276.1:CH/ZMDZY/11/China-2011*	GII-C2	−	−	−	+	+	+	+
23	KF761675.1:CH/YNKM-8/China-2013	GII-C3	MH726398.1:GDS37/China-2011	GII-C9	KX812523.1:XM1-2/China-2016	GII-C1	−	−	−	+	+	+	−
24	JQ023161.1:Virulent DR13/South Korea-2009	**GI-a**	EF185992.1:LZC/China-2007*	**GI-a**	KC109141.1:JS2008-2/China-2012*	**GI-b**	−	−	−	−	−	+	−
25	MK644604.1:N7-GD2017/China-2017	GII-C6	MH726392.1:GDS44/China-2013	GII-C8	MK796238.1:CN/Liaoning25/2018/China-2018	GII-C3	−	−	−	−	−	+	−
26	MH061342.1:CH/SCZJ/2018/China-2018	GII-C3	MH107321.1:GDS10/China-2013*	GII-C2	MK409659.1:ZJ15XS0101-P120/China-2016*	GII-C1	−	−	−	−	−	+	−
27	KM609208.1:PEDV-15F/China-2012	GII-C3	MH061342.1:CH/SCZJ/2018/China-2018	GII-C3	MK409659.1:ZJ15XS0101-P120/China-2016	GII-C1	−	−	−	−	−	+	−
28	MK644605.1:T10-HB2018/China-2018	GII-C9	MH061341.1:CH/SCLS/2018/China-2018	GII-C9	KM609212.1:PEDV-LYG/China-2014	GII-C1	−	−	−	−	−	+	

The potential recombination events were identified by each of 7 algorithms (RDP, GENECONV, Bootscan, MaxChi, Chimaera, SiScan, and PhylPro) embedded in the RDP4 package. Genogroup was defined based on the phylogenetic tree of full-length spike gene of the PEDVs in this study (see Figure 1).

R: RDP; G: GENECONV; B: BootScan; M: MaxChi; C: Chimaera; S: SiScan; T: Phylpro. +: verified; −: not verified.

^*^ The major or minor parent may be the actual recombinant due to the possibility of misidentification.

The recombination events occurred mainly in between the PEDVs in GII genogroup (Table 1). Three GI-a viruses are involved in recombination (KX839247.1:85–7-mutant1/China-2015 in event 2; JQ023161.1: Virulent DR13/South Korea-2009 and EF185992.1:LZC/China-2007 in event 24), whereas two GI-b viruses (MN315264.1: AH-2018-HF1/China-2018 in event 5 and KC109141.1:JS2008-2/China-2012 in event 24) are found to participate in the recombination (Table 1, Figure 2). We also find that 20 PEDV isolates seem to be involved in multiple recombination events (Table 2).

**Table 2:** **The PEDVs isolated in China and involved in multiple recombination events in the full-length spike genes**

GenBank ID: Virus name/country-year	Recombination Event serial number (see Table 1)	GenBank ID: Virus name/country-year	Recombination Event serial number (see Table 1)
MN486588.1:Ah2016f2/China-2016	Event 2, 8, 14	MH581489.1:CH/HBTS/2017/China-2017	Event 15, 21
MK140813.1:CH/TP-4–3/2018/China-2018	Event 4, 11	MH061342.1:CH/SCZJ/2018/China-2018	Event 26, 27
MK138516.1:V7-HB2018/China-2018	Event 5, 15	KM609208.1:PEDV-15F/China-2012	Event 18, 19, 27
KM609204.1:PEDV-7C/China-2011	Event 8, 10, 12, 13	MH726406.1:GDS49/China-2017	Event 1, 6, 7, 13, 16
MH726374.1:GDS11/China-2014	Event 8, 10	MH061337.1:CH/SCZG/China-2017	Event 4, 13
KM609209.1:PEDV-CHZ/China-2013	Event 7, 9, 11, 14	MH726401.1:GDS41/China-2011	Event 3, 22
KM609203.1:PEDV-1C/China-2012	Event 4, 17	MH056657.1:JSX2014/ATT/China-2018	Event 10, 12
MK644601.1:G2-HE2017/China-2017	Event 11, 15	MK409659.1:ZJ15XS0101-P120/China-2016	Event 16, 26, 27
MN037494.1:WHLL/China-2017	Event 19, 21	KX812523.1:XM1-2/China-2016	Event 20, 23
KF761675.1:CH/YNKM-8/China-2013	Event 2, 23	MK409659.1:ZJ15XS0101-P120/China-2016	Event 26, 27

As shown in Figure 2, in 19 out of 28 recombination events, both beginning and ending breakpoints are located in 5’- or 3’-proximal regions of S gene, which encode the N-terminus or C-terminus of S protein, respectively. In 9 recombination events, i.e. events 15, 16, 18, 20, 21, 23, 24, 26, and 28, the breakpoints are located in the middle region of S gene.

**Figure 3:**
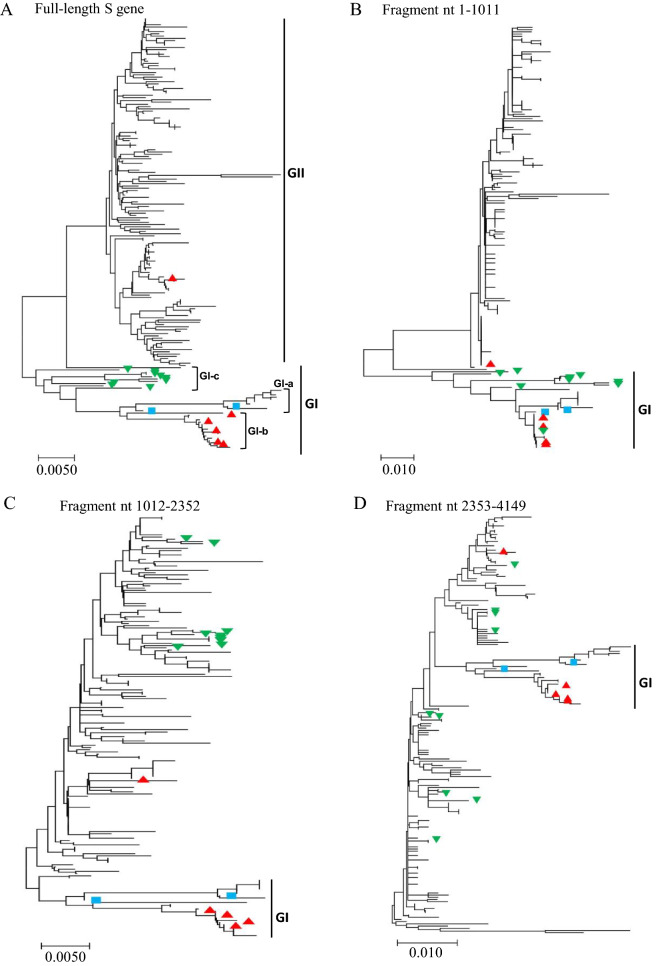
**The phylogenetic trees based on the full-length or indicated fragments of PEDV S genes that have been analyzed in Figure ****1****.** The blue squares indicate the virulent strains isolated outside of China. The red triangles indicate the cell culture-adapted vaccine strains or the pandemic strains used to make vaccines. The green upside-down triangles indicate the GI-c viral isolates.

To further explore the recombination of PEDV S gene, the phylogenetic trees were constructed based on the individual gene fragments that encode different domain of S protein. The fragment nt 1–1011 (relative to the S gene of PEDV virulent CV777, GenBank: AF353511.1) encodes D0 and about 100-amino acids N-terminus of S1 domain of S protein. The fragment nt 1012–2352 encodes mainly the larger part of S1 domain, whereas the fragment nt 2353–4149 encodes the remaining part of S protein including S2 domain, transmembrane domain (TM), and a short cytoplasmic tail (CT) (Figure 2A). As shown in Figure 3, the phylogenetic trees based on the full-length S gene or individual gene fragments are not superposable to each other. For example, compared with full-length S gene-based phylogenetic tree (Figure 3A), the fragment nt 1–1011 of the members of GI-c subgroup (indicated with the green upside-down triangles) are genetically closer to that of virulent viruses (CV777 and DR13; indicated with the blue squares) and 6 vaccine variants (indicated with the red triangles; Figure 3B). However, the fragment nt 1012–2352 (Figure 3C) and fragment nt 2353–4149 (Figure 3D) of the members of GI-c subgroup are genetically far more distant from that of virulent or vaccine variants. These results are reminiscent of the observation that S gene recombination between GI-a/GI-b and GII variants may result in the emergence of some GI-c viruses.

### Variations in linear B cell epitopes of PEDV S protein

**Figure 4:**
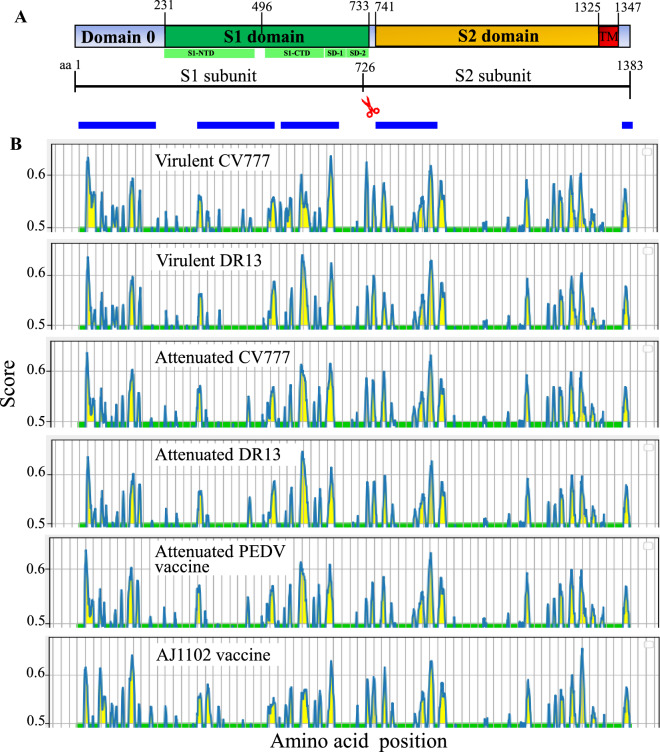
**Linear B cell epitope map of full-length S protein of indicated PEDVs.** **A** Diagram depicting the main features of the PEDV S protein, including: putative cleavage site between S1 and S2 subunits at aa 726, signal peptide (aa 1–18), Domain 0 (aa 19–230), and the transmembrane domain (TM, aa 1325–1347). Functionally conserved S1 domain is at aa 231–733, whereas the S2 domain at aa 741–1344. Four subdomains are found in S1 domain, which include S1-NTD (S1 N-terminal domain, aa 231–471), S1-CTD (S1 C-terminal domain, aa 504–637), and Subdomains 1 and 2 (SD-1 and SD-2, aa 638–733). The numbers indicate the amino acid position relative to the S protein of virulent CV777 (GenBank ID: AF353511.1). The blue blocks represent the relative positions of protein regions where the neutralizing epitopes have been experimentally verified. **B** The linear B cell epitope map was obtained by using BepiPred-2.0 server. The amino acid residues with scores above the threshold value that was set at 0.5 were predicted to be part of an epitope and colored in yellow on the graph. Y-axes depicts residue scores and X-axes indicates amino acid positions in the sequence, which is also relative to the diagram on the top (**A**).

Analysis of S protein sequence of PEDV prototype virulent CV777 in NCBI conserved domain database (CDD) [69–72]) reveals that the S1 subunit contains a conserved S1 domain at aa 231–733, whereas the S2 subunit contains a conserved S2 domain at aa 741–1344 (Figure 4A). The S1 domain can be further divided into four subdomains including S1-NTD (S1 N-terminal domain, aa 231–471), S1-CTD (S1 C-terminal domain, aa 504–637), and Subdomains 1 and 2 (SD-1 and SD-2, aa 638–733) [24]. The Domain 0 in S1 subunit is located upstream of S1 domain [24], where no conserved motifs were detected in the CDD (Figure 4A).

Sequence comparison of the full-length S proteins reveals that GI-a, GI-b, and GI-c strains share 99%, 96%, and 95% of amino acid sequence with prototype strain CV777 respectively, but GII strains share only 93% (Table 3), indicating that GII strains are less closely related to that in GI genogroup. Further amino acid sequence comparison of individual regions of S protein demonstrates that Domain 0 of S protein in GI-a, GI-b, GI-c, and GII exhibit 98%, 96%, 93%, and 85% of identities with that of prototype CV777 (Table 3). When compared with the identities for S1 domain or S2 domain, Domain 0 of S protein is the most variable, particularly for that from GII genogroup (Table 3).

**Table 3:** **Pairwise comparison of the amino acid sequences of full-length S protein or its individual domains including Domain 0, S1 and S2 domains from GI-a, GI-b, GI-c, and GII genogroup with that of PEDV reference strain virulent CV777 (GenBank ID: AF353511.1)**

Query: virulent CV777	Full-length	Domain 0	S1 domain	S2 domain
GI-a (*n* = 9)	99.17 ± 0.29	98.23 ± 0.54	99.48 ± 0.42	99.29 ± 0.24
GI-b (*n* = 16)	96.77 ± 0.18	96.03 ± 0.07	96.50 ± 0.26	97.30 ± 0.17
GI-c (*n* = 9)	95.13 ± 0.32	93.07 ± 1.61	95.01 ± 0.15	95.99 ± 0.32
GII (*n* = 155)	93.79 ± 0.49	85.41 ± 0.86	94.97 ± 0.43	95.97 ± 0.68

The percent amino acid identity (%) was presented as the mean ± standard deviation (SD).

**Figure 5:**
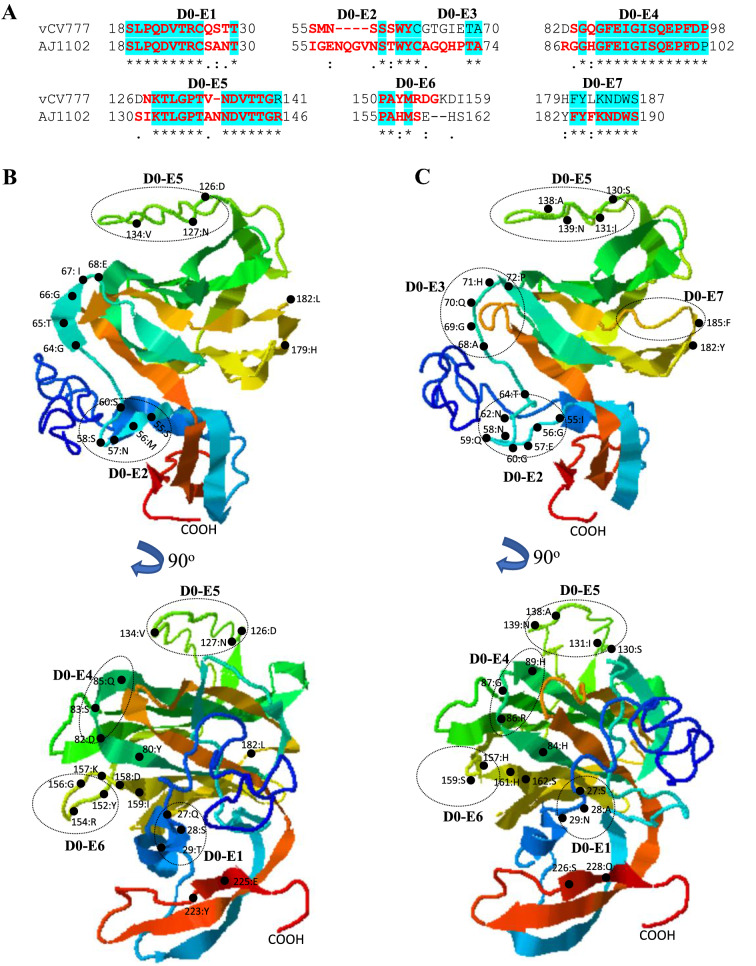
**Comparison of linear B cell epitopes affected by amino acid variations in the Domain 0 of S proteins between GI-a strain virulent CV777 and GII strain AJ1102**. **A** The amino acid sequence of predicted linear B cell epitopes are shown with red bold letters. The numbers indicate the amino acid positions relative to the full-length S protein of indicated PEDVs. **B** 3-dimentional view of epitopes in the Domain 0 of virulent CV777 spike protein. **C** 3-dimentional view of epitopes in the Domain 0 of AJ1102 spike protein. The tertiary structure was modeled by using I-TASER. The individual epitope is circled and the amino acid variations are labeled with black dot. vCV777, virulent CV777.

PEDV AJ1102 (GenBank:JX188454) is a GII-C8 strain that was isolated in a PEDV epidemic in China in 2011 [73]. This strain is currently being developed into a potential vaccine [50]. To explore the potential effects of amino acid variations on the antigenicity of S protein, we compare the linear B cell epitopes of S proteins between 6 PEDV strains from GI and GII genogroups. The BepiPred-2.0 server was used to predict the potential linear B cell epitopes [62]. As shown in Figure 4B, the full-length S proteins from GI-a (virulent CV777 and DR13) and G1-b (attenuated CV777 and DR13) subgroups exhibit the similar distribution pattern of predicted B cell epitopes. However, the Domain 0 of GII genogroup strain (AJ1102) shows the most obvious differences in distribution of B cell epitopes when compared with other virus strains (Figure 4B). Furthermore, we used I-TASER server [63–65] to visualize the 3-dimentional structures for Domain 0, S1 and S2 domains, where the amino acid variations in the B cell epitopes will be structurally compared. As shown in Figure 5A, the Domain 0 of AJ1102 S protein contains a 4-amino acids insertion (58NQGV61), a 1-amino acid insertion (139 N), and a 2-amino acids deletion (in between aa160-161) when compared with that of prototype virulent CV777. This insertion-deletion genetic signature of S protein as S INDEL (S insertion-deletion) has been well known for PEDV strains [43, 74]. Those variations are all located in the sequence of predicted epitopes (Figures 5A–C). The mutation of 5 consecutive amino acids (68AGQHP72) and one amino acid mutation (185F) lead to the generation of novel potential epitopes in AJ1102 S protein (D0-E3 and D0-E7, respectively) (Figures 5A–C).

**Figure 6:**
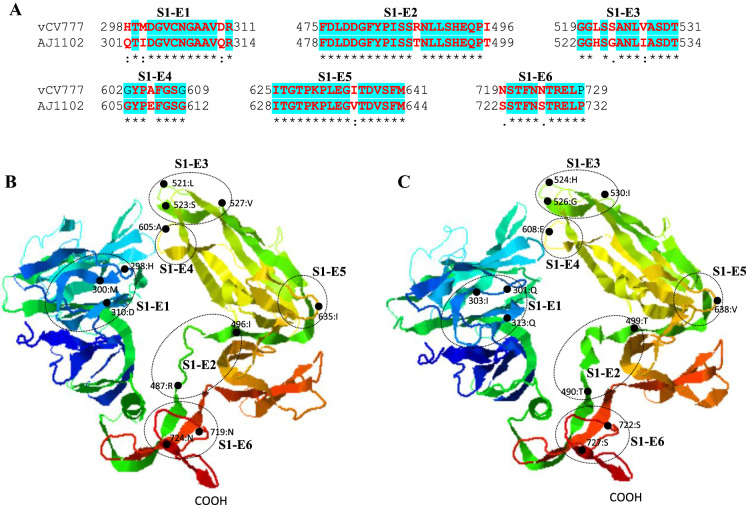
**Comparison of linear B cell epitopes affected by amino acid variations in the S1 domain of S proteins between GI-a strain virulent CV777 and GII strain AJ1102**. **A** The amino acid sequence of predicted linear B cell epitopes are shown with red bold letters. The numbers indicate the amino acid positions relative to the full-length S protein of indicated PEDVs. **B** 3-dimentional view of epitopes in the S1 domain of virulent CV777 spike protein. **C** 3-dimentional view of epitopes in the S1 domain of AJ1102 spike protein. The individual epitope is circled and the amino acid variations are labeled with black dot. vCV777, virulent CV777.

When compared with prototype strain virulent CV777, the amino acid variations in S1 domain of AJ1102 are found in 6 potential epitopes, but does not generate novel epitopes nor eliminate the existing ones (Figure 6A). All those epitopes are located in the coiled region or at the end of β-sheets (Figure 6B). S1-E6 has been experimentally confirmed to be a linear neutralizing epitope [36].

**Figure 7:**
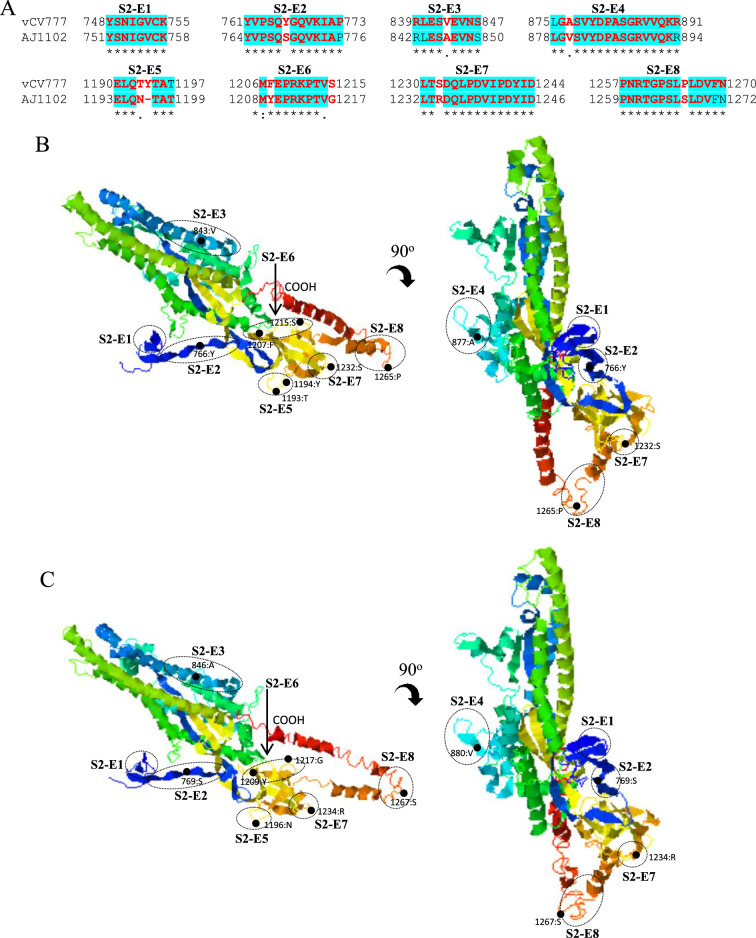
**Comparison of linear B cell epitopes affected by amino acid variations in the S2 domain of S proteins between GI-a strain virulent CV777 and GII strain AJ1102**. **A** The amino acid sequence of predicted linear B cell epitopes are shown with red bold letters. The numbers indicate the amino acid positions relative to the full-length S protein of indicated PEDVs. **B** 3-dimentional view of epitopes in the S2 domain of virulent CV777 spike protein. **C** 3-dimentional view of epitopes in the S2 domain of AJ1102 spike protein. The individual epitope is circled and the amino acid variations are labeled with black dot. vCV777, virulent CV777.

In S2 domain of PEDV S protein, the predicted B cell epitopes S2-E1 to S2-E4 are located at the N-terminal region, whereas epitopes S2-E5 to S2-E8 at the C-terminal region (Figures 7A and B). 7 of total 8 epitopes are found to be affected by amino acid variations (S2-E2 to E8). The epitope S2-E1 is identical between virulent CV777 and AJ1102 strains. This epitope has been experimentally confirmed to be a linear neutralizing epitope [36]. Collectively, the hypervariable Domain 0 of S protein (Figures 4 and 5) may represent one of the major factors driving the antigenic drift of S protein, which may contribute largely to the ineffectiveness of GI genogroup-based vaccines including attenuated PEDV prototype CV777 in protecting piglets from the GII genogroup virus-caused epidemics in China since 2010 [3, 53, 55]. This situation poses a major challenge to the prevention and control of PED in China.

## Discussion

The S protein of PEDV plays critical roles in virus infection. It recognizes the cellular receptor via specific interactions during virus entry into the host cells and elicits the production of neutralizing antibodies. The S protein gene was also used to determine the genetic diversity of viral isolates. To explore the genetic relationships and evolution of PEDVs that circulated in China during 2007–2019, the complete nucleotide and deduced amino acid sequences of a total of 186 full-length S genes were analyzed in this report. Wang et al. [50] previously performed phylogenetic analysis of 62 PEDV strains isolated in China during 2010–2015 on the basis of full-length genomic sequences and sorted those virus strains into two genogroups, GI (classical) and GII (field variant). Each genogroup has two sub-groups: GI-a and GI-b in GI genogroup, GII-a and GII-b in GII genogroup [50]. GI-a subgroup consists of the Chinese classical strains (LZC, CH/S, and CHM2013), along with the prototype strains (virulent CV777 and DR13), whereas GI-b subgroup predominantly contains the cell-culture-adapted vaccine strains (attenuated CV777 and DR13) and other pandemic classical strains (AH-M, SD-M, SQ2014, and SC1402) [50]. The most prevalent strains isolated from outbreaks during 2010–2015 belong to the GII genogroup [50]. In our study, 124 more PEDV strains including 60 viruses isolated in China during 2015–2019 were included in the analysis. In agreement to Wang et al.’s report [50], most PEDVs isolated from outbreaks after 2015 (but not including 2015) in China belong to the GII genogroup (Figure 1). Only one viral isolate (MN315264.1:AH-2018-HF1/China-2018) is sorted into GI-b subgroup, whereas no new viral isolate belongs to GI-a subgroup (Figure 1). Additionally, 9 PEDVs isolated during 2016–2018 emerged as a new subgroup in GI genogroup (GI-c), in which virus TW/Yunlin550 has been characterized in a recent report [68]. For GII genogroup, the PEDVs can be further sorted into 10 clusters in our study instead of two subgroups (GII-a and GII-b) classification used by Wang et al. [50].

In this study, we found that the S gene of PEDVs in China is characterized by high level of the recombination. A total of 28 potential recombination events was found in the S genes (Figure 2, Table 1, and Table 2). The recombination occurred predominantly between GII genogroup PEDVs, indicating that GII genogroup viruses remain the major threat to the pig farming industry in China. The recombination not only drives the expansion of GII genogroup, but also serves as a critical factor to boost the generation of novel evolutionary lineages, which were classified into GI-c subgroup and resulted from the genetic exchanges between GII variants and GI-a or GI-b variants (Table 1, Figures 2, 3). The emergence of these new recombinants makes the field pandemics more complicated and heterogenous in China, posing extra challenges to the development of effective prevention strategies. The infectivity and pathogenicity of the GI-c recombinants have yet to be explored.

The S protein is a major antigen eliciting the production of neutralizing antibodies against PEDV. Several regions of the S protein have been found to contain neutralizing epitopes. Li et al. reported that the Domain 0 of the S protein can elicit neutralizing antibodies [22]. This region is also responsible for the virus binding to the sialic acid during virus entry [75]. Amino acid sequence comparison between GII stain AJ1102 and GI-a prototype virulent CV777 reveals that the antigenicity of this region seems to be mostly varied as amino acid variations dramatically affected the predicted linear epitopes (Figures 4 and 5). The amino acid variations were also found in 13 predicted epitopes in S1 and S2 domains (Figures 6 and 7, respectively). The epitopes S1-E3, S1-E4, and S1-E5 are located in a neutralizing epitope-containing subdomain [76]. S1-E6, S2-E1, and S2-E2 are located in a region spanning the S1/ S2 junction of PEDV S protein, to which a series of monoclonal antibodies (mAbs) with neutralizing activity have been identified including SD37-11, SD20-1, SD121-1, SD129-3, SD129-5, SD131-3, SD137-90, SD138-108, and SD142-2 [37]. Collectively, these results suggest that the variations in GII S protein may compromise or even eliminate the neutralizing activities of antibodies elicited by GI S proteins in traditional vaccine strains used in China, which is consistent with the phenomenon that the GI-derived vaccines failed to provide effective protection of piglets from prevailing GII variants-dominated infections since 2010 in China [38, 42, 43, 77, 78]. Therefore, the GII-based vaccines should be given the priority for pig-farming industry in China.

In summary, the study of S genes and proteins of PEDVs circulating during 2007–2019 in this report demonstrates that GII genogroup and GI-c subgroup represent two major targets for the development of effective prevention strategies in China. The information presented here would benefit the design of novel vaccines for PEDVs circulating in China.

## Supplementary Information

**Additional file 1.** **The phylogenetic tree based on the full‐length spike gene of porcine epidemic diarrhea viruses (PEDVs) isolated in China during 2007–2019 (Continue on Fig. ****1****).**

## Data Availability

The nucleotide and protein sequence data used in this study are available in GenBank.
