# Supplementary figures and images for: The spike glycoprotein genes of porcine epidemic diarrhea viruses isolated in China
Source: Vet Res. 2021 Jun 15;52:87. doi: 10.1186/s13567-021-00954-6 (PMC8205199; doi:10.1186/s13567-021-00954-6)

## Slide 1
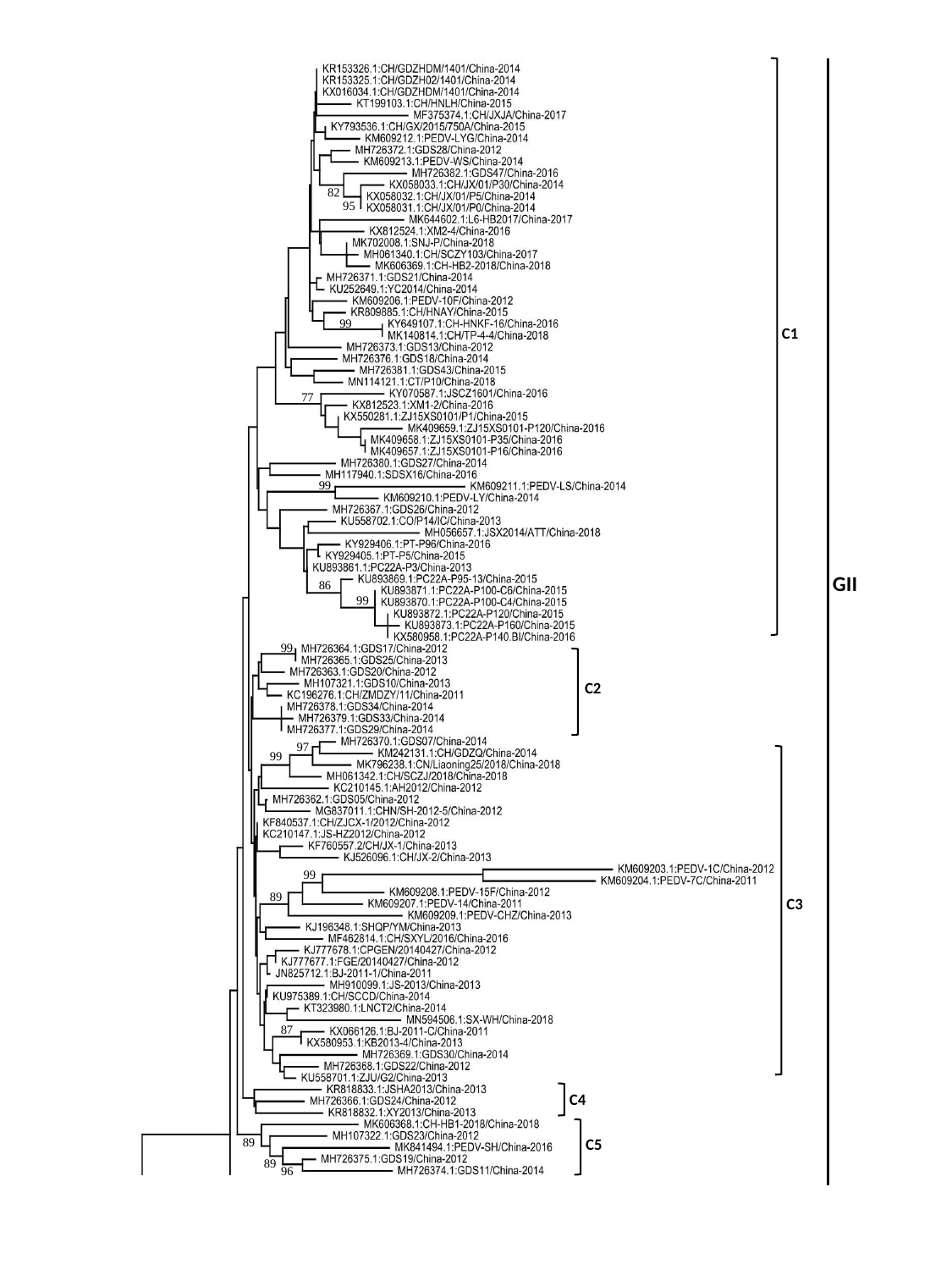

82
95
99
C1
77
99
GII
86
99
99
C2
97
99
99
89
C3
87
C4
89
C5
89
96

Supplement: Supplementary file 1 — Additional file 1. The phylogenetic tree based on the full‐length spike gene of porcine epidemic diarrhea viruses (PEDVs) isolated in China during 2007–2019 (Continue on Fig. 1). [file 13567_2021_954_MOESM1_ESM.pptx]
